# Beyond the clinic: sensory-adapted food services to support nutrition in autism spectrum disorder

**DOI:** 10.1017/S1368980026102420

**Published:** 2026-04-08

**Authors:** Maria Vittoria Conti, Lauren Fiechtner, Hellas Cena

**Affiliations:** 1 University of Pavia, Pavia, Italy; 2 General Academic Pediatrics and Pediatric Gastroenterology, Mass General Hospital for Children, Boston, USA; 3 ICS Maugeri IRCCS, Pavia, Italy

**Keywords:** Autism spectrum disorder, Food selectivity, Nutrition, Malnutrition, Public services

## Abstract

Autism spectrum disorder (ASD) is a population-scale condition with life-course health consequences, yet nutrition support remains inconsistently embedded in routine pathways. Food selectivity is common in ASD and is associated with restricted dietary variety, nutritional imbalance, gastrointestinal morbidity and cardiometabolic vulnerability. Current responses are predominantly clinic-and family-centred and are difficult to scale equitably. This commentary argues that institutional food services (schools, day-care and residential settings) are an underused public health platform to improve inclusion and accountability through sensory-accessible, nutritionally adequate meals. Because these services are commissioned, standardised and audited, sensory accessibility can be operationalised via procurement specifications and quality indicators, enabling benchmarking across sites. Evidence from sensory-informed menu adaptation and implementation work suggests feasibility within routine operations and supports evaluation using system-relevant outcomes (acceptability, nutritional adequacy, waste, feasibility and maintenance). Three policy actions are proposed: embed sensory accessibility in institutional standards, integrate nutrition across sectors and fund scale-up using implementation science.

Autism spectrum disorder (ASD) is a population-scale condition with life-course health consequences that extend beyond specialist neurodevelopmental services^(1,2)^. Yet nutrition – despite its importance for health, functioning and participation – remains inconsistently embedded in routine support across childhood, adolescence and adulthood.

Food selectivity is not a behavioural footnote; it is a health, inclusion and equity issue. In public systems, nutrition can function as a practical lever for inclusion and accountability: it determines whether everyday services deliver equitable access to acceptable, nutritionally adequate meals. Restricted food repertoires and sensory-driven preferences are more common in people with ASD than in neurotypical peers and are associated with lower dietary variety and heightened risk of nutritional imbalance^(3–5)^. These patterns intersect with gastrointestinal morbidity and cardiometabolic vulnerability^(6–8)^. The consequences are tangible: family burden, reduced participation in shared meals and avoidable inequities in institutional settings – schools, day-care services and residential facilities – where meals structure daily life.

The dominant response to food selectivity has been clinic-and family-centred. Behavioural feeding interventions and caregiver-led strategies can be effective, but they are difficult to scale, unevenly accessible and frequently disconnected across health, education and social care^(9–12)^. A public health approach must therefore ask a different question: where can nutrition support reach people with ASD daily, at scale, with measurable accountability?

Institutional food services are a high-reach platform. Institutional meals shape exposure and norms through repetition, default options and social routines – features that can either widen gaps (when menus are inaccessible) or reduce them (when systems are designed for inclusion). Standard ‘healthy menus’ can fail in ASD when sensory accessibility is ignored: nutritionally adequate foods may be refused, worsening nutritional risk and increasing waste. In this context, food selectivity becomes a modifiable interface between sensory profiles and food systems – an interface that institutions can redesign. Because these services are already governed through procurement and quality standards, sensory accessibility can be operationalised as a measurable component of routine public-services performance.

This makes institutional meals an unusually scalable public health lever: unlike clinic-based interventions, food services are commissioned, standardised, audited and budgeted. Embedding sensory accessibility into procurement specifications and quality indicators would therefore translate neurodiversity inclusion into enforceable service requirements – what is served, what is accepted and what is wasted – rather than relying on individual advocacy. In practice, this shifts nutrition support from discretionary ‘extra care’ to routine performance management, enabling benchmarking across sites and reducing postcode inequities.

A setting-based model has been developed and tested for institutional food services that combines sensory-informed menu engineering (texture, temperature, odour intensity and visual complexity), predictable meal presentation and delivery, and training and support for staff and caregivers. Early evidence indicates this approach can produce system-relevant outcomes that matter to public services: acceptability, nutritional adequacy, feasibility for staff workflows and indicators of efficiency such as reduced waste.

In the FOOD-AUT pilot study in an adult day-care setting, tailored menu adaptations aligned with nutritional adequacy and sensory preferences improved meal acceptance, demonstrating feasibility within routine food service operations^(13)^. This work was translated into practical, implementation-oriented recommendations for adapting institutional menus, turning sensory accessibility into actionable catering specifications rather than leaving acceptability to chance^(12,13)^.

The AUT-MENU project extended this approach across multiple sites and wider age ranges under real-world constraints. Baseline assessments confirmed substantial heterogeneity across centres – exactly the context in which scalable public health strategies must operate^(14)^. Menu adaptations were well tolerated and improved the nutritional profile of meals without compromising acceptability, even when total consumption did not change significantly^(15)^. This distinction is critical for scale: in settings where dietary variety cannot be rapidly expanded, improving nutritional quality within accepted sensory boundaries may represent a pragmatic first step towards better life-course health trajectories. AUT-MENU also integrates caregiver-focused nutrition education and provides a protocol for broader implementation and evaluation in routine services^(14)^.

The public health implications are direct. Sensory-adapted catering can reduce inequities by delivering consistent, evidence-informed nutritional support independent of family resources, local specialist availability or individual advocacy capacity. It also creates accountability pathways: institutions can measure and report outcomes such as acceptability, nutritional adequacy, waste, feasibility and maintenance – metrics routinely used to govern quality and performance in public services. Moving from promising projects to policy requires implementation science, with explicit specification of implementation outcomes and evaluation frameworks to support adoption, fidelity and sustainability across heterogeneous services^(16–18)^.

Three policy actions could accelerate translation.

1. Make sensory-accessible nutrition a standard in institutional meals. Procurement and food service guidelines could incorporate sensory accessibility alongside nutritional standards, supported by staff training, practical menu specifications and routine monitoring of consumption and waste.
2. Integrate nutrition into ASD pathways across sectors. Nutrition support could be embedded in clinical follow-up but operationalised where meals occur – in schools and social care services – through shared standards that bridge systems without medicalising everyday life.
3. Fund and evaluate scale-up, not only pilots. Hybrid effectiveness-implementation designs, equity metrics and longer-term outcomes are needed to establish what works, for whom and under what conditions^(16–18)^.

Food selectivity in ASD demands system-level solutions. An institutional sensory-adapted model – supported by staff and caregiver training and evaluated with implementation science – offers a pragmatic pathway from clinics to communities. Redesigning institutional meals to be both nutritionally adequate and sensory-accessible is not a clinical luxury; it is a public health responsibility towards neurodiverse citizens and a test of how systems deliver inclusion through everyday infrastructures^(12–15)^.
